# Dermatophytic Biofilms: Characteristics, Significance and Treatment Approaches

**DOI:** 10.3390/jof9020228

**Published:** 2023-02-09

**Authors:** Anthi-Marina Markantonatou, Konstantinos Samaras, Timoleon-Achilleas Vyzantiadis

**Affiliations:** Department of Microbiology, Medical School, Aristotle University of Thessaloniki, 541 24 Thessaloniki, Greece

**Keywords:** dermatophytes, dermatophytic infections, biofilms, antifungal susceptibility testing

## Abstract

Microbes are found in the environment, possibly more often as biofilms than in planktonic forms. Biofilm formation has been described for several important fungal species. The presence of a dermatophytoma in a dermatophytic nail infection was the basis for the proposal that dermatophytes form biofilms as well. This could explain treatment failure and recurrent dermatophytic infections. Several investigators have performed in vitro and ex vivo experiments to study the formation of biofilms by dermatophytes and their properties. The nature of the biofilm structure itself contributes to fungal protection mechanisms against many harmful external agents, including antifungals. Thus, a different approach should be carried out regarding susceptibility testing and treatment. Concerning susceptibility testing, methods to evaluate either the inhibition of biofilm formation, or the ability to eradicate it, have been introduced. As for treatment, in addition to classical antifungal agents, some natural formulations, such as plant extracts or biosurfactants, and alternative approaches, such as photodynamic therapy, have been proposed. Studies that connect the results of the in vitro and ex vivo experimentation with clinical outcomes are required in order to verify the efficacy of these approaches in clinical practice.

## 1. Introduction

Dermatophytes are pathogens exhibiting tropism for tissues rich in keratin, such as skin, nails and hair. They may affect both humans and animals, and they can also be found in soil where they use decomposing keratinous materials for nutrition. According to the latest taxonomy by De Hoog et al., dermatophytes are currently classified into seven Clades that represent the following seven genera: *Trichophyton*; *Epidermophyton*; *Nannizzia*; *Paraphyton*; *Lophophyton*; *Microsporum;* and *Arthroderma* [1]. Dermatophytic infections are quite common in the general population and the majority of them are superficial. Their severity varies from mild to quite severe cases. Although they are not life threatening, they may compromise the individual’s quality of life because their symptoms affect physical and, in certain cases, psychological health. Empirical treatment, namely treatment based on prior experience or even without the identification of the pathogen and the performance of susceptibility testing, is common practice. Additionally, in many cases, it seems to be effective. However, there are cases of chronic or recurrent dermatophytoses that require a more specialized approach [2,3]. The application of antifungal susceptibility testing (AFST) could be useful in such cases. Still, dermatophytes’ special characteristics, such as a slow growth rate and poor sporulation, complicate the method and may delay the results. Some investigators have suggested modifications to facilitate the procedure [4,5,6,7], whereas the EUCAST recently published official guidelines for the susceptibility testing of microconidia-forming dermatophytes [8].

Some rational explanations about the persistence of dermatophytic infections and resistance to treatment could be poor compliance to treatment because long-term therapies are often required (even three to twelve months for a nail infection) or pharmacokinetic issues regarding the ability of the drug to reach infected tissues. First, Burkhart et al. in 2002 described a dermatophytoma that could be the cause of resistance to treatment and introduced the idea of dermatophytic biofilms [9]. Biofilms are structures consisting of microbial cells and an extracellular matrix, and they behave in a completely different way than planktonic cells. Bacterial biofilms have been thoroughly studied, as well as those produced by potentially lethal fungal pathogens, such as *Candida* and *Aspergillus* spp. Burkhart introduced the suggestion of their existence also in dermatophytic fungal infections. 

Subsequently, the aim of this work is to present the current knowledge about dermatophytic biofilms and their properties, as well as the existing approaches of their susceptibility testing and treatment. In order to achieve this, a review of the literature was attempted. The relevant articles were retracted through Pubmed and Scopus, and they concern the time interval from 2001 to present.

## 2. Biofilms and Dermatophytes

Microbes are found in the environment more as biofilms than in planktonic forms [10,11]. The latter is used to describe free cells suspended in a solvent, whereas biofilms are more complicated structures consisting of large groups of cells and an extracellular matrix (ECM). The formation of biofilms was first observed in bacteria, and it has also been described for several important fungal species, including *Candida* [12], *Cryptococcus* [13], *Aspergillus* [14], *Trichosporon* [15], *Pneumocystis* [16] and *Coccidioides* [17]. In 2002, Burkhart et al. suggested that the formation of a dermatophytoma (a circumscribed fungal mass inside the nail) was an indication that dermatophytes also have the ability to form biofilms [9].

The first step in the biofilm formation procedure is the adhesion of fungal cells on a suitable substrate, which may be either a mucosal or abiotic surface. These cells proliferate, form hyphal structures and subsequently (as the biofilm matures) produce an extracellular matrix which is composed of polysaccharides, lipids, proteins and nucleic acids [18,19]. Fungi, exactly as the other microbes, prefer this kind of growth because it provides them plenty of advantages. Every living organism needs nutrients and protection against external dangers in order to survive, while biofilm structures fulfill both these conditions. In terms of protection, fungal elements enclosed into the biofilm are protected against the action of the host immune system and antifungal agents [20,21,22]. In addition, the extracellular matrix facilitates the diffusion, the use of nutrients and the cooperation between the cells [23,24].

In 2014, Costa-Orlandi et al. first described the characteristics of dermatophytic biofilms according to their in vitro studies on *Trichophyton rubrum* and *Trichophyton mentagrophytes* reference strains [25]. Sterilized coverslips were used as abiotic substrates, and after a 3 h pre-adhesion phase, the fungal inoculum was incubated without agitation at 37 °C for 72 h. Subsequently, non-adherent cells were removed, and the morphology was studied by light microscopy. The quantification of the biofilm mass and the extracellular matrix was performed by the use of crystal violet and safranin staining, respectively. The XTT reduction assay was used to determine the metabolic activity, whereas scanning electron microscopy (SEM) and confocal laser-scanning microscopy (CLSM) were used for further studies of the biofilm morphology. These studies confirmed that both species exhibit the ability to form biofilms, but *T. rubrum* produced a denser mass with higher biomass and extracellular matrix production.

In 2017, Toukabri et al. performed susceptibility studies using clinical isolates from patients with foot mycosis, including 21 *T. rubrum* and 5 *Trichophyton interdigitale*, alongside with non-dermatophytic molds [26]. During these studies, all the aforementioned strains exhibited the ability to form biofilms. 

In addition to previous in vitro studies, Brilhante et al. performed ex vivo studies on nail fragments to evaluate the biofilm-forming ability of various dermatophytic isolates (*T. rubrum*, *Trichophyton tonsurans*, *T. mentagrophytes*, *Microsporum canis* and *Microsporum gypseum*) [27]. The vast majority of them were capable of forming biofilms either in vitro or ex vivo, with the exception of two *M. canis* strains. *Microsporum gypseum*, *T. rubrum* and *T. tonsurans* were characterized as strong biofilm producers with higher biomass in contrast to *T. mentagrophytes* and *M. canis*. The latter seems to produce the weakest biofilms in comparison to the other species. Based on previous studies, the authors assumed that this fact could be explained by differences in cell adhesion [28] and in the type or number of enzymes that different species produce [29]. Another remarkable result was that *T. rubrum* produced higher biomass ex vivo (on nail fragments) than in vitro. Scanning electron microscopy revealed interesting details about the three-dimensional structure of the biofilms. Those formed in vitro contained hyphae that were grew in several directions, whereas the extracellular matrix filled the area between hyphae. In the ex vivo model, the fungal elements and the extracellular matrix replaced the nail surface in some parts of the nail. 

Ex vivo studies have also been performed on animal hair (from cats and dogs) to prove the biofilm-forming ability of *M. canis*, *M. gypseum*, *T. mentagrophytes* and *T. tonsurans* strains [30]. All the species were able to produce biofilms within a time interval of 14 days, whereas microscopy revealed degradation of the hair shaft, mycelial growth and conidia production, alongside the abundant production of extracellular matrix.

## 3. Resistance to Antifungals

Antifungal drug resistance is a serious problem regarding the treatment of invasive fungal infections; however, it is also an emerging problem for dermatophytic infections. Dermatophytes try to confront antifungal agents, using several different mechanisms of resistance, such as drug degradation, the overexpression of genes, mutations in targeted genes and multi-drug efflux transporters [31]. Biofilm formation is an additional and very important resistance mechanism. 

The nature of the biofilm structure itself contributes to the related microorganisms’ protection mechanisms against any harmful external agent, including antifungals. Studies performed on *C. albicans* [32] and *A. fumigatus* [14] (two common fungal pathogens) have demonstrated the important role of the extracellular matrix and persister cells. The extracellular matrix acts as a barrier to antifungal agents, whereas the drug–polysaccharide interaction may affect the effectiveness of the drug [14]. As for persister cells, they are highly tolerant and probably dormant [33] cells that survive from the action of antifungals and have the ability to proliferate when the drug is no longer present in their environment [34]. It has been shown that *C. albicans* biofilms contain persister cells that are responsible for multi-drug tolerance [32]. Additionally, the upregulation of genes related to multi-drug resistance has been demonstrated for both *A. fumigatus* and *C. albicans* [35,36], as well as for genes involved in ergosterol biosynthesis [35,36,37]. 

Another study on *C. albicans* demonstrated that the glucan produced is used not only for cell wall formation, but that it is also incorporated into the extracellular matrix and provides the ability to sequestrate the antifungals. Thus, it prevents drugs from reaching their target, resulting in the survival of the fungal cells even at very high exposure levels [38]. 

As far as dermatophytes are concerned, after Burkhart’s suggestion on the presence of relevant biofilms (dermatophytoma) as a cause of resistance to treatment, many investigators have tried to introduce suitable methods of susceptibility testing in order to also take biofilm formation into account.

## 4. Susceptibility Testing 

The EUCAST recently provided guidelines about antifungal susceptibility testing in dermatophytes [8], whereas the CLSI incorporated to its guidelines instructions about dermatophytic molds several years ago [39]. Both methods are suitable for testing susceptibility to antifungals with regards to dermatophytes in their planktonic form. Bearing in mind all the aforementioned special characteristics of biofilms, it is obvious that there should be a different approach with regards to susceptibility testing and treatment. Concerning susceptibility testing, there have been methods introduced to evaluate either the inhibition of biofilm formation or the ability to eradicate them [26,40,41,42,43,44,45,46,47,48,49,50,51,52,53,54,55,56]. Usually, the concentrations able to inhibit or eradicate biofilm structures are much higher than that of common MICs that are measured by assays using planktonic cells [42,50,57]. 

All the suggested in vitro assays use polystyrene plates and an RPMI 1640 culture medium, while incubation is performed at 37 °C. There is an initial pre-adhesion phase during which the fungal inoculum is transferred into the plates and, after a certain incubation period (usually 3 h), aspiration and washing take place in order to remove any non-adherent cells. Subsequently, antifungal substances are added to the wells and their effect on biofilm formation is evaluated up to 72 h later. To collect information about the effect of antifungals on mature biofilms, the drugs are added after 72 h of incubation and the estimation is conducted 24 h after the addition of them. 

Analyses of the biofilms are performed by the use of various techniques and substances. Regarding the in vitro experiments, XTT-tetrazolium salt [40,42,45,46,48,51] and MTT-tetrazolium dye [41,52,53] are used to perform colorimetric techniques that provide indirect indications about the metabolic activity and, subsequently, the viability of the biofilms. Crystal violet [26,43,47,49,50,52,53] and safranin [26,44,54] are also used for colorimetric techniques that yield biomass quantification. Scanning electron microscopy (SEM), confocal laser-scanning microscopy (CLSM) and fluorescence microscopy are used for both in vitro and ex vivo experiments [44,45,46,47,48,49,50,51,52,53,54,55,56], and they provide information about the biofilm structure. The addition of sterile water to the wells with the preformed biofilms, the suspension of their cells and subsequent quantification of the CFUs after inoculation in Sabouraud dextrose agar is a method that is also applied in both in vitro and ex vivo experiments [43,45,48,55]. In all cases, positive and negative controls are used, and all the aforementioned methods are evaluated by comparing the wells under examination with the control wells.

All the aforementioned antibiofilm susceptibility testing studies are summarized in Table 1.

## 5. Treatment Potentials

The antifungals mostly used to treat dermatophytoses are allylamines, azoles and griseofulvin, either in topical or systemic administration [57]. The susceptibility studies described previously have mainly tested terbinafine, as well as griseofulvin, fluconazole, itraconazole, voriconazole, econazole and ciclopirox additionally. Griseofulvin was also tested in a chloroform solvate, as well as in non-solvated dispersions. Both exhibited a significant reduction in biofilm formation [44]. Recently, Rocha et al. tried an interesting combination of the antidepressant sertraline with the antifungal caspofungin. Their studies demonstrated that there was an excellent synergistic effect [52].

Additionally, some natural formulations were tried, such as plant extracts. Hydroxychavicol (a phenolic component of *Piper betle*, a plant that usually grows in Southeast Asia) [40], isoflavaspidic acid PB (derived from *D. fragrans*, a plant species native to tropical Africa) [46] and farnesol (a 15-carbon acyclic sesquiterpene alcohol originally extracted from Farnese acacia tree *Vachellia farnesiane*) [56] were tested in vitro with favorable results. Hydroxychavicol proved to be effective against dermatophytosis as well, as was demonstrated by studies on an animal model [40]. Additionally, some biosurfactants, such as the sophorolipid produced by *Rhodotorula babjevae* [47], a rhamnolipid (a biosurfactant which contains rhamnose) produced by *Pseudomonas aeruginosa* [54], as well as a biosurfactant produced by the entomopathogenic fungus *Beauveria bassiana* [48], were effective in vitro against dermatophytic biofilms. The sophorolipid was also tested on an in vivo mouse model with favorable results [47], whereas the biosurfactant produced by *Beauveria bassiana* was effective against ex vivo formed biofilms on healthy hair [48]. Veiga et al. tested propolis ethanol extract, a resin-like material produced by bees, which demonstrated in vitro antifungal activity against planktonic cells, as well as biofilms [43]. The ex vivo study showed good penetration of propolis through the nails, whereas a clinical investigation of 16 patients demonstrated excellent clinical improvement.

Other substances, such as various antidandruff formulations, were also tested [41] and some of them exhibited antibiofilm activity. In 2020, Costa-Orlandi et al. studied the efficacy of the substance nonyl 3, 4-dihydroxybenzoate incorporated into nanostructured lipid systems [55] in order to avoid the toxicity of high concentrations. The formulation proved to be effective against *T. rubrum* and *T. mentagrophytes* biofilms. Recently, Brilhante et al. studied the antibiofilm effects of Proteinase K, a broad-spectrum serine protease, and showed that it has antibiofilm properties and a synergistic effect when combined with antifungals [53]. 

In a review on antibiofilm treatment for onychomycoses, Gupta proposed that biofilms should be disrupted prior to antifungal treatment [2]. In order to achieve this disruption, the components of the extracellular matrix, namely polysaccharides, proteins and extracellular nucleic acids, should be targeted. The enzymes deoxyribonuclease I (DNase I), α-amylase and lyase have been tested, as well as other substances, such as lactic acid, chitosan, terpinen-4-ol-loaded lipid nanoparticles and povidone-iodine (PVP-I) [2]. All the above have been tested in bacterial biofilms and were effective in certain microorganisms [58,59,60,61,62,63,64,65,66]. Specifically for fungal biofilms, DNase demonstrated favorable results in combination with amphotericin B [67] and terpinen-4-ol-loaded lipid nanoparticles were very effective against *C. albicans* biofilms [68]. As far as dermatophytes are concerned, Brilhante et al. [53] recently explored the inhibitory effect of proteinase K against dermatophytic biofilms and showed that it causes a reduction in mature biofilms. Following biofilm disruption, further techniques in combination with antifungal agents could be applied. 

Taraszkievicz et al. proposed the use of photodynamic therapy (PDT) by the use of a LED light source [69], which has also been demonstrated to be effective against *C. albicans* biofilms [70,71]. Chen et al. showed that PDT seems to make dermatophytic biofilms more susceptible to antifungals [45], whereas Bila et al. tried the substance 2-chalcone (a flavonoid precursor) as a photosensitizer for PDT with favorable results against biofilms formed by *T. rubrum* and *T. mentagrophytes* strains [51]. Another interesting alternative approach is that of surface acoustic waves (SAWs), which have been tested and proved to be effective on bacterial biofilms in combination with antibacterial drugs [72].

## 6. Discussion

Dermatophytic infections, although not life threatening, often cause considerable issues in patients. Sometimes they are difficult to treat or concern recurrent infections. This may happen due to patients’ poor compliance to treatment owing to the regimen’s long duration, the emerging problem of infections by resistant strains or otherwise due to specific patient characteristics (differences in the composition of keratin among individuals that make dermatophytes host specific [73], defects of phagocytes [3], CARD9 deficiency [74] and individuals with a defective skew to Th2 immunity that may exhibit a predisposition to chronic dermatophytic infections [73]). Furthermore, a novel, highly virulent species has emerged. *T. indotineae* exhibits considerable resistance to terbinafine causing dermatophytic infections that are very difficult to treat, often with large lesions [75]. Biofilm formation also seems to play an important role. Initial information about biofilms was obtained by studies on bacteria and subsequent studies on potentially life-threatening fungal pathogens, such as *Candida* and *Aspergillus*. The results of these studies were used to approach dermatophytic biofilms, as well due to their main characteristics (way of formation and basic properties) that are common. The most interesting part is their ability to remain highly resistant to treatment. Two main characteristics that could play an important role are the capturing of the antifungals from the extracellular matrix and the presence of persister cells. Brilhante et al. studied in vitro and ex vivo biofilm formation and noticed that *T. rubrum* had the ability to produce much higher biomass in comparison with the other analyzed species. They assumed that this amount of biomass represented the development of a hyphal network surrounded by the extracellular matrix and that it could be a rational explanation regarding the difficulty in treating *T. rubrum* infections [27].

Although several in vitro and ex vivo experiments have demonstrated the formation of dermatophytic biofilms, in vivo visualization has not yet been achieved. Visualization on skin biopsies is not applicable because the structure collapses during the dehydration process [76]. Their definite presence in onychomycoses has also not been proved. However, there are indications, such as the dermatophytes’ in vitro and ex vivo ability to form biofilms, the latter’s firm adherence to the nail plate and their resistance to treatment, that make the presence of dermatophytic biofilms in onychomycoses quite plausible. As for skin infections, scanning electron microscopy has revealed bacterial biofilms in chronic wounds [77]; thus, it could be possible to also have dermatophytic biofilms.

Antifungal susceptibility testing can offer valuable information to optimize treatment. However, classical AFST methods use planktonic suspensions, which do not represent the exact nature of the fungal elements inside the infected lesions. It has been demonstrated that when AFST is performed by the use of biofilm structures, there is up to a 50-fold increase in MICs [42,50]. Given that certain species, such as *T. rubrum*, have the ability to produce large amounts of biofilm material [25,27], the performance of AFST on biofilms becomes necessary. Several investigators have proposed methods to determine MICs in dermatophytic biofilm formations. Beside the conventional antifungals, natural products have been tested with satisfying results. Plant derivatives, various biosurfactants produced by bacteria or fungi and bee products, such as propolis, have been tested. As it has previously happened, these natural products could be the starting point for the discovery of new drugs that could be effective against dermatophytic biofilms. Additionally, substances already tested and proved to have antifungal properties have been tested in different formulations, such as nonyl 3,4-dihydroxybenzoate, which have been incorporated into a nanostructured lipid system [55]. Recent studies on an antidepressant (sertraline), which proved to act synergistically with the antifungal caspofungin against the dermatophyte *T. rubrum,* were also interesting [52].

A very interesting approach to treatment is the proposal to act step by step: first to disrupt the biofilm structure, and then to administrate the main antifungal to kill the cells that live inside the structure. Disruption may be accomplished by the use of chemical or natural methods. Chemicals target certain components of the extracellular matrix, whereas natural approaches use light (PDT) or surface acoustic waves (SAWs) to decompose the structure and make the cells inside it vulnerable to classical antifungals. Regarding photodynamic therapy, a natural photosensitizer (the flavonoid precursor 2-chalcone) has shown to be effective against dermatophytes [51]. The advantage of these approaches is that lower dosages of antifungals are required, and thus toxicity and side effects are reduced, as well as the probability of drug resistance [78]. However, these applications do not affect persister cells, which on the other hand could be possibly targeted with certain dosing schemas that would attack them when they try to “wake up” and multiply.

In addition to in vitro studies, many investigators performed ex vivo experiments on human hair and nails as well as on Persian cat hair, to study the dermatophytes’ behavior and their interaction with antifungal substances [48,49,52]. Additionally, in vivo studies were performed on animal models. Ali et al. studied tinea corporis (induced by *T. mentagrophytes var. interdigitale*) [40] in guinea pigs, whereas Sen et al. used a mouse model of cutaneous dermatophytosis (induced by *T. mentagrophytes*) to estimate the therapeutic efficiency of the biosurfactant sophorolipid [47]. In 2018, a clinical investigation was performed by Veiga et al. [43]. The study included 16 patients with onychomycosis who were treated with propolis ethanol extract with excellent correspondence.

## 7. Conclusions

Dermatophytes form biofilms in order to favor their survival. This characteristic is a major reason that makes the treatment of dermatophytic infections in some cases quite difficult. As far as we know, the potential of biofilm formation by the highly *virulent T. indotineae* species has not yet been explored. Future studies in this field could be very useful in terms of further understanding the infection’s pathophysiology and if biofilm formation is verified for appropriate applications of antibiofilm susceptibility testing. Furthermore, new drugs or even classical antifungals, in combination with novel mechanical methods, could contribute and give a new perspective to the treatment of complicated cases. Additional studies that connect the results of in vitro and ex vivo experiments on dermatophytic biofilms with clinical outcome are required in order to verify the efficacy of all the aforementioned approaches in clinical practice.

## Figures and Tables

**Table 1:** Summary of the antibiofilm susceptibility testing studies on dermatophytes, including the dermatophytic strains, methods used and the main conclusions.

Study No.	Ref. No.	Author/Year	Strains	Αntifungal Agents	Methods	Conclusions
1	[41]	dos Santos et al., 2015	*T. mentagrophytes* ATCC 9533, *T. rubrum* ATCC 28189	5 antidandruff formulations	MTT viability staining	- Inhibition of fungal growth, variable efficacy - 2/5 formulations were effective against mature biofilms
2	[40]	Ali et al., 2016	- 40 dermatophytic isolates (clinical isolates and 1 reference strain of each species: *E. floccosum* MTCC 613, *M. gypseum* MTCC 2819, *T. mentagrophytes* ATCC 9533, *T.rubrum* MTCC 296) - in vivo studies on guinea pigs (tinea corporis)	Hydroxychavicol (major phenolic component of *Piper betle*)	Biofilm formation quantified by XTT reduction assay	- All isolates susceptible to hydroxychavicol - Inhibitory effect on biofilm formation and reduction in preformed biofilms of *T. mentagrophytes* reference strain
3	[26]	Toukabri et al., 2017	- 26 clinical isolates: 21 *T. rubrum* and 5 *T. interdigitale* (tinea pedis, onychomycosis)	FLC, ECO, ITR, TRB, GRF	Biofilm quantification by crystal violet and safranin red	TRB the most effective antifungal against *T.rubrum* and *T. interdigitale*
4	[42]	Brilhante et al., 2018	23 clinical isolates: 4 *T. rubrum*, 6 *T. tonsurans*, 3 *T. mentagrophytes*, 7 *M. canis*, 3 *M. gypseum*	ITR, VRC, GRF	- Biofilm biomass quantification by crystal violet - XTT assay to estimate the efficacy against mature biofilms	Biofilm cells more tolerant to antifungals than their planktonic forms, in both weak and strong biofilm-forming isolates
5	[43]	Veiga et al., 2018	- 45 clinical isolates (onychomycosis): 29 *T. rubrum*, 13 *T. mentagrophytes*, 2 *T. verrucosum*, 1 *T. interdigitale* - 2 strains (1 *T. rubrum*, 1 *T. interdigitale*) for the antibiofilm assays - ex vivo studies in nail fragments to evaluate the propolis extract penetration into the nail - clinical investigation in 16 patients	Propolis ethanol extract (PE)	- Quantifying the number of CFUs and biomass - Biofilm biomass quantification by crystal violet	- In vitro antifungal activity in both planktonic cells and preformed biofilms - PE able to penetrate through the nail - Excellent clinical improvement
6	[44]	Al-Obaidi et al., 2019	1 *T. rubrum* isolate	Griseofulvin solvate solid dispersions	- Biofilm visualization with CLSM - Biofilm quantification by safranin staining - Metabolic activity by XTT assay	Griseofulvin solvate exhibits significantly higher antifungal activity in comparison to non- solvated form
7	[45]	Chen et al., 2019	19 clinical isolates (onychomycosis): 6 *T. rubrum*, 10 *T. mentagrophytes,* 3 *M. gypseum*	TRB, ITR, CPX, FLC alone or in combination with photodynamic treatment	- Biofilm structures studied with SEM - Quantification of biofilm formation and determination of MICs by XTT assay - CFU counting	- Highly efficient photodynamic inhibition/ CFU reduction - Biofilms become more susceptible to antifungals after PDT
8	[46]	Lin et al., 2019	20 clinical isolates (10 *T. rubrum*, 6 *T. mentagrophytes*, 2 *M. canis*, 2 *M. gypseum*) and 5 standard strains (2 *T. rubrum*, 1 *T. mentagrophytes*, 1 *M. canis*, 1 *M. gypseum*)	- Isoflavaspidic acid PB (extracted *from D. fragrans*) - AmpB, TRB as reference antifungal agents	- XTT assay for effects on biofilm formation and on preformed biofilms - SEM for the morphology of mature biofilms - Biomass analyzed by gravimetric analysis - Extracellular exopolysaccharide quantification with anthranone sulfuric acid method - Ergosterol content of *T. rubrum* mature biofilms by UPLC	- All isolates effectively inhibited - TRB the most effective antifungal agent - Isoflavaspidic acid PB can inhibit biofilm formation; destroy pre-mature biofilms; and decrease biofilm biomass, biofilm extracellular exopolysaccharide and ergosterol content
9	[47]	Sen et al., 2020	- 1 *T. mentagrophytes* strain - in vivo studies in a mouse model	- Sophorolipid produced by *Rhodotorula babjevae* - TRB as a standard drug	- Mycelia studied by SEM - Mycelial topography (visualization of the effect) by AFM and CLSM	- Effective against planktonic forms and biofilms - In vivo therapeutic efficiency
10	[48]	Abdel-Aziz et al., 2020	- *M. canis* ATCC 36299 - ex vivo on healthy hair	A biosurfactant produced by *Beauveria bassiana* (BBLP)	- XTT assay to measure the inhibitory effects on biofilms - ex vivo biofilms were visualized using stereo microscopy, SEM and FM - CFU counting	- Mycelial growth of *M. canis* significantly inhibited - The biofilm eradication percentage increased when increasing the BBLP concentration - Effective against ex vivo biofilms
11	[49]	Castelo-Branco et al., 2020	- 18 dermatophytic strains (12 *M. canis*, 6 *T. mentagrophytes*) - ex vivo model on Persian cat hair	GRF, ITR, TRB	- Biofilm biomass quantification by crystal violet - Metabolic activity by XTT reduction assay - SEM and CLSM for the evaluation of the ex vivo biofilm susceptibility testing	- Dermatophytic biofilms grown in vitro are more tolerant to antifungals (higher MICs) in comparison to planktonic growth - Dermatophytic biofilms grown ex vivo are more tolerant to antifungals than those grown in vitro
12	[54]	Sen et al., 2020	1 *T. rubrum*, 1 *T. mentagrophytes*	rhamnolipid (RL-SS14) produced by *Pseudomonas aeruginosa*	- Crystal violet staining - ECM quantification by safranin staining - Biofilm dispersal ability estimated by crystal violet and safranin staining - Biofilm imaging by SEM, CLSM and AFM	- Inhibitory and disruptive effects on biofilms
13	[55]	Costa-Orlandi et al., 2020	*T. rubrum* ATCC 28189, *T. rubrum* ATCC MYA-4438 and *T. mentagrophytes* ATCC 11481	Nonyl 3,4-dihydroxybenzoate incorporated into a nanostructured lipid system	- Metabolic activity estimated by XTT reduction assay - Topographical characteristics analyzed by SEM - CFU counting	- The incorporation into the lipid system maintains its effectiveness against both planktonic cells and biofilms
14	[56]	Brilhante 2021	Chlamydoconidium-producing *Trichophyton tonsurans* strains	TRB and farnesol	- Analysis of metabolic activity - Quantification of biomass - Observation by SEM	- Both TRB and farnesol reduced biomass and metabolic activity of mature biofilms (64.4–69%)
15	[51]	Bila et al., 2021	*T. rubrum* ATCC 28189, *T. rubrum* ATCC MYA-4438, *T.mentagrophytes* ATCC 11481	2-chalcone (flavonoid precursor), TRB, FLC	- Metabolic activity by XTT reduction assay - Biofilms’ topographic analysis by SEM - Evaluation of cell damage by CLSM	- 2-chalcone: anti-dermatophytic and antibiofilm properties - 2-chalcone’s use as a photosensitizer for PDT is effective against dermatophytes
16	[52]	Rocha et al., 2022	- 1 *T. rubrum* strain - ex vivo study (human nail infection assay)	Sertraline with or without caspofungin	- Metabolic activity by MTT assay - Biofilm quantification by crystal violet - Visualization by SEM	- Excellent synergistic effects (in vitro inhibition of *T. rubrum*) - Low concentrations of sertraline are effective against *T. rubrum* biofilms if combined with caspofungin
17	[53]	Brilhante et al., 2022	14 dermatophytic strains: (3 *M. canis*, 5 *T. tonsurans*, 4 *T. mentagrophytes*, 1 *T. rubrum*, 1 *E. floccosum*)	Proteinase K, TRB, GRF	- Biofilm biomass quantification by crystal violet - Biofilm metabolic activity by MTT - Visualization by CLSM+ Live/Dead fluorescent dye (SYTO9/propidium iodide) and SEM	- Proteinase K causes a reduction in the mature biofilms - Synergistic effect against mature biofilms when combined with antifungals
18	[50]	Yazdanpanah et al., 2022	50 clinical isolates of nail, skin and hair infections: 14 *T. mentagrophytes*; 13 *T. rubrum*; 4 *T. interdigitale*; 2 *T. tonsurans*; 2 *T. verrucosum*; 1 *T. persicum*; 1 *T. simii*; 6 *M. canis*; 3 E. *floccosum*; 1 *Nannizzia gypsea*; 3 unknown	TRB, GRF, ITR	- Biofilm quantification by crystal violet - Visualization of the ECM by optical microscopy - Visualization of the biofilms’ three-dimensional structure by SEM	- A high percentage (74%) of the clinical isolates were capable to form biofilms in vitro - TRB had excellent inhibitory activity against biofilm formation - Concentration needed for 80% prevention of the biofilm formation was up to eightfold of the TRB MIC

FLC: fluconazole; ECO: econazole; ITR: itraconazole; TRB: terbinafine; GRF: griseofulvin; VRC: voriconazole; CPX: ciclopirox; AmpB: amphotericin B; CLSM: confocal laser-scanning microscopy; SEM: scanning electron microscopy; AFM: atomic force microscopy; FM: fluorescence microscopy; UPLC: ultra-performance liquid chromatography; ECM: extracellular matrix, PDT: photodynamic therapy, CFUs: colony-forming units.

## Data Availability

No new data were created or analyzed in this study. Data sharing is not applicable to this article.
